# Geometric features of pig airways using computed tomography

**DOI:** 10.14814/phy2.12995

**Published:** 2016-10-24

**Authors:** Md K. Azad, Hansen A. Mansy, Peshala T. Gamage

**Affiliations:** ^1^ Biomedical Acoustics Research LaboratoryDepartment of Mechanical and Aerospace EngineeringUniversity of Central FloridaOrlandoFlorida; ^2^ Rush University Medical CenterChicagoIllinois

**Keywords:** Airway tree, bifurcating plane, generation, image segmentation, transition zone

## Abstract

Accurate knowledge of the airway geometry is needed when constructing physical models of the airway tree and for numerical modeling of flow or sound propagation in the airways. Human and animal experiments are conducted to validate these models. Many studies documented the geometric details of the human airways. However, information about the geometry of pig airways is scarcer. Earlier studies suggested that the morphology of animal airways can be significantly different from that of humans. The objective of this study is to measure the airway diameter, length and bifurcation angles in domestic pigs using computed tomography. In this study, lungs of six pigs were imaged, then segmentation software tools were used to extract the geometry of the airway lumen. The airway dimensions were measured from the resulting 3‐D models for the first 24 airway generations. Results showed that the size and morphology of the airways of the six pigs were similar. The trachea diameters were found to be comparable to the typical human adult, but the diameter, length and branching angles of other airways were noticeably different from that of humans. For example, pig airways consistently had an early branching from the trachea that feeds the top right lung lobe and precedes the main carina. This branch is absent in the human airways. The results suggested that the pig airways geometry may not be accurately approximated by human airways and this approximation may contribute to increasing the errors in computational models of the pig chest.

## Background

### Objectives

Lung sounds are often used for diagnosis of pulmonary conditions. Several studies suggested the utility of computerized acquisition of these sounds (Mansy et al. 2002b; O'Connor et al. 2005; Dellinger et al. 2008; Pasterkamp et al. 1997). Relevant acoustic phenomenon in the pulmonary system have been studied using animal (Kraman and Wang 1990; Mansy et al. 2002a; Räsänen et al. 2014) benchtop (Acikgoz et al. 2008; Dai et al. 2015; Mansy et al. 2015b) and numerical (Royston et al. 1999, 2002; Zhang et al. 2001; Ozer et al. 2007) models. To validate computational models, animal experiments can be carried out (Dai et al. 2014a,b; Henry et al. 2014; Peng et al. 2014, 2015). Accurate information on animal airway geometry would be necessary for building realistic models of the airways. The purpose of this study was to document the geometry of the airways in domestic pigs using computed tomography (CT). The geometric details of interest include airway diameter, length, bifurcation angles, radius of curvature of branches, and relations between consecutive bifurcating planes. This information can be used to build realistic geometries for computer models of the acoustic transmission in the pig airways.

### Available information on airway tree

Human airway geometry have been documented by many studies (Horsfield and Cumming 1967, 1968; Horsfield et al. 1971), while some studies discussed the airway geometry in dogs, rat, sheep and hamster (Yeh et al. 1976; Tawhai et al. 2004). On the other hand, information about the pig airway geometry is more scarce or incomplete (Maina and Gils 2001; Monteiro et al. 2014). In addition, the animal airway geometry can be significantly different from that of humans. Hence, using human airway geometry as an approximation for pig geometry in computational models can introduce errors.

### Airway classification methods

Airways can be categorized by generations (Weibel 1963) and/or ordering schemes (Strahler 1957; Horsfield and Cumming 1968; Horsfield et al. 1971). As an example, Weibel (1963) classified the airways by assigning each airway a generation number starting at the trachea (generation zero) which is increased by one at each branching. In this approach, symmetric bifurcations were assumed, where each parent airway branches into two identical twins that have a higher generation.

On the other hand, Horsfield (Horsfield and Cumming 1968; Horsfield et al. 1971) adopted an ordering method, where the peripheral conducting airways are given order 1 and the order increases by one at each bifurcation up from the peripheral airways towards the trachea. Strahler (1957) proposed a similar ordering scheme, where the order of the parent branch is one order higher than its two children branches of same order. However, if the two children benches have different orders, the parent branch order is the same as the child branch with the higher order. Since the latter two ordering schemes start the numbering system at the terminal airways, they ideally require a tree that contains at least some of these branches.

In this study, we chose to assign generation numbers starting from the trachea since the available airway tree deals with relatively larger airways that did not contain the terminal bronchioles.

## Methods

### Imaging of the airways

The lungs and airways were extracted from six domestic pigs (weight ~40 kg) after Institutional Animal Care and Use Committee (IACUC) approval. Power calculations were used to help choose the sample size. This analysis indicated that a sample size of six was needed assuming a power of 0.75, a level of confidence of 0.85 and a standard deviation to be about 0.75 of the effect. In this study, a relatively small sample size was preferred due to the pilot nature of the study and the high cost associated with the experiments. Lungs were preserved using a propriety method (Bio Quest LS03765; Nasco, Fort Atkinson, WI) that allows easily re‐inflating the airways. In preparation for performing CT, lungs were inflated at 20 cm water for 1 h after intubation with an endotracheal tube. The lungs were imaged using a CT scanner (Brilliance 64; Phillips Healthcare, Andover, MA). The resolution of the CT data was 512 × 512 pixels (pixel size = 0.8 mm) with axial step of 1 mm and the scan covered the region extending from the proximal trachea to the distal lung. CT exams were performed at Rush University Medical Center, Chicago, IL.

### Airway segmentation

The CT scan data files were processed using a segmentation shareware program (ITK‐SNAP, PICSL, University of Pennsylvania & SCI, University of Utah) to create 3‐D models (Yushkevich et al. 2006) from 3D images. The program implements a “volume filling algorithm” that relies on voxel intensity to find the airways. The steps used during segmentation include:

1. Select a region of interest and an automatic segmentation mode (called “Active Contour Segmentation”).
2. Classify voxels into background (dark) and foreground (light) groups using intensity thresholding (by choosing the “region of competition” feature). Set the region competition force to 1.000 and the smoothing force to 0.001. These parameters control how the “snake” algorithm will fit the boundary of the foreground/background regions.
3. Place “bubbles” inside some of the airways of the region of interest. These bubbles are the seeds where automatic segmentation will begin and grow these bubbles (like a snake inside a hollow tube). The radii of the bubbles are user adjustable, and were chosen to be slightly smaller and completely inside of the airways to help prevent “leakage” of the volume filling to outside the airways during the segmentation process. More than one bubble can be placed to speed up auto‐segmentation.
4. Control the segmentation speed by selecting a step size. A step size of 1 was used in this study to allow close monitoring of the volume filling algorithm results, although that would correspond to the slowest segmentation. The segmentation was stopped around 1500 iteration steps to decrease the likelihood of leakage.
5. After automatic segmentation is complete, the voxels of the smaller airways that were not detected by the program were labeled manually. This step allowed the user to fill individual voxels using a cursor. The user would rely on the voxel color to choose the voxels that belong to inside the airways. In the CT scans of this study, the voxels inside the airways were dark, whereas airway walls were lighter. Hence, the voxels that were dominantly dark were labeled as part of the airways. Some of the dark gray voxels were filled to create airways with a smoothed lumen.

### Airway dimensions

Two different approaches were applied to measure the diameter, length, and bifurcation angles for proximal airways (found by automatic and manual segmentation). In the first method, a physical model was built by 3D printing (Stratasys Dimension Elite, Eden Prairie, MN, 0.178–0.254 mm layer thickness) of the segmented airways. A digital caliper (resolution = 0.01 mm) was used to measure the airway dimensions and angles directly. In the second method, computer software (AutoCAD 2012; Autodesk, San Rafael, CA) was used to rotate the computer model of the airways in space and measure the dimensions using the software “dimension tool.” In this method, the output of the segmentation program was converted using a mesh processing software (MeshLab, 1.3.3; Visual Computing Laboratory, Pisa, Italy) to “DXF” file format to be easily read by AutoCAD.

To label the airways, they were first arranged according to their diameter from larger to smaller. The trachea was labeled as the first generation (generation 0) and smaller airways are progressively given higher generation numbers. When the airways had similar diameters (within 0.3 mm) they were assigned the same generation numbers since the uncertainty in diameter measurements was estimated to be approximately 0.3 mm. This approach extends the methods suggested in earlier studies (Weibel 1963) in order to account for nonsymmetric bifurcations. This modification in the method was necessary because the majority of the pig airway bifurcations in this study are not symmetric.

## Results

### Segmentation

**Figure 1:**
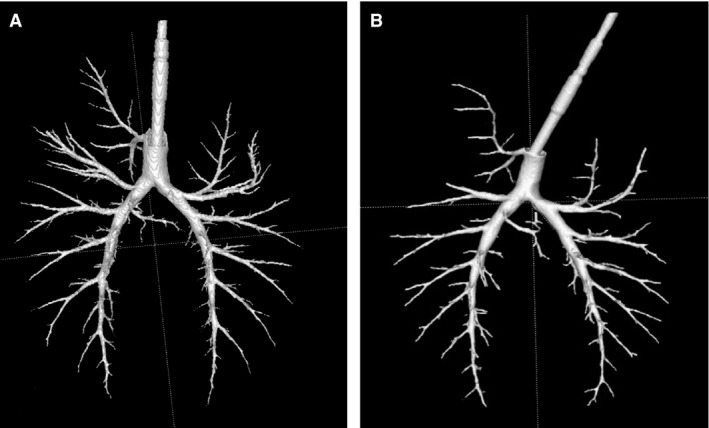
Example of airways that were extracted from CT images of two different pigs (A. Pig 2 and B. Pig 3) using automatic and manual segmentation. The airways are viewed from the anterior side. There is an early airway branching that feeds the top right lung lobe, which was consistently seen in the six study subjects but not usually seen in humans. In addition, the two main bronchi tended to have monopodial morphology and continue in the same general direction after several branching.

The lungs and airways of six pigs were acquired and imaged using CT. Then the airway geometry was extracted from the CT scans using ITK‐SNAP software. Segmentation parameters were chosen to maximize accuracy of airway labeling. This also ensured optimization of the number of airways that can be automatically found while avoiding “leakage” outside the airways. The process consisted of two main steps. First, the images were analyzed by inspecting the histogram of the image intensity; then a linear transformation of the intensity values was performed to enhance image contrast such that the whole intensity range was covered. The same transformation parameters were applied to all CT slices. Next, multiple bubbles (typically, six bubbles with diameter = 2 mm) were placed in the airways and the volume filling function (called “snake” in the software) was utilized to grow the bubbles to label the airways. The airways found by this automatic segmentation varied in size with the smallest diameter of about 2 mm. Automatic segmentation typically took 2 min and resulted in about 60 branches. It was followed by manual segmentation where voxels were manually labeled. Both automatic and manual segmentation were performed with input from healthcare personnel experienced in reading CT. Manual segmentation typically took 8 h to label about 60 small airways, with a typical diameter between 3 and 1 mm. This article focuses on the results of the automatic and manual segmentation. Figure 1 shows an example of the airway structures extracted using automatic and manual segmentation (Fig. 1).

### Airway dimensions

**Figure 2:**
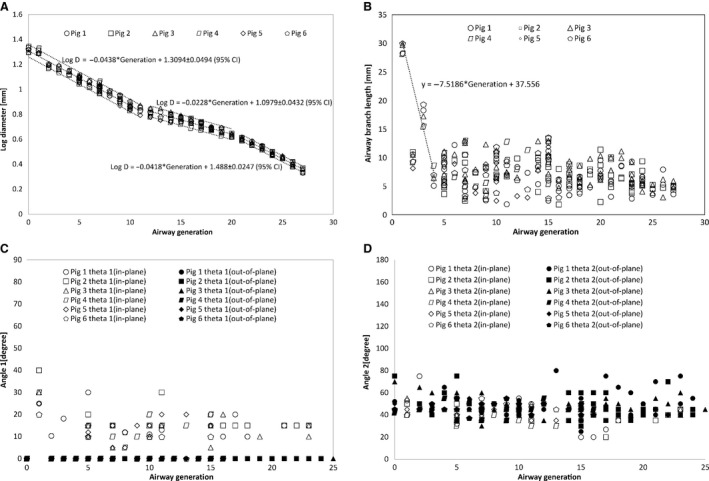
(A) Semi log graph of airway diameter versus generation. (B) Airway length versus generation. (C) Bifurcation angle for major daughter (angle 1) versus generation. (D) Bifurcation angle for minor daughter (angle 2) versus generation.

In this study, airway dimensions and bifurcation angles were initially measured using two different methods (measuring dimensions of a computer model or a 3D printed model). Since differences between the two methods were small (±0.3 mm, *P* < 0.05, *t*‐test) only one method was used for the rest of the pigs. Figure 2 shows the log airway inner diameter, length, and bifurcation angles as a function of the parent generation. The trends for airway diameters, lengths, and angles were similar among individual animal subjects. The log diameter versus airway generation plot showed three different trends as airway generation increased. The logarithm of the diameter was found to be linearly related to generations and dropped relatively steep in the early generation up to generation 11 and can be expressed by the equation Log D = −0.0438 × Generation + 1.3094 ± 0.0494 (95% confidence interval). This was followed by a less steep drop up to generation 20 which can be expressed as Log D = −0.0228 × Generation + 1.0979 ± 0.0432 (95% confidence interval). Finally, it starts to drop sharper at higher generations with the following expression, Log D = −0.0418 × Generation + 1.488 ± 0.0247 (95% confidence interval). The airway branch length versus generation plot shows approximately linear trend up to generation 4 with the exception of generation 2 (right mainstem) where the length was relatively short. At higher generations, the length varied between 5 and 12 mm without a clear trend. In an asymmetric bifurcation, a parent branch bifurcates into two daughters of dissimilar diameters. This study showed that this bifurcation can happen in two different planes. In the first case, the parent branch, major (larger) daughter and minor (smaller) daughter stayed approximately in the same plane as the two main stem bronchi, which can be described as in‐plane bifurcation. In the second case, the parent branch and major daughter stayed in the same plane, whereas the minor daughter bifurcated into a plane approximately perpendicular to the plane of the parent and major daughter. This branching trend can be called out‐of‐plane bifurcation. This study found that the diameters and bifurcation angles for out of plane bifurcation were different than in‐plane bifurcation cases. Figure 2C shows the bifurcation angle between a parent branch and major daughter (angle 1) versus airway generation for in‐plane and out‐of‐plane bifurcations. The figure shows that most of the angle 1 values for the out‐of‐plane bifurcations were almost zero while for in‐plane bifurcation, angle 1 values were in the range 10–20 degree. The mean angle 1 for in‐plane bifurcation was 14.34 degree with a 95% confidence interval of 1.56 degree. This suggests a small change in the major daughter (daughter 1) direction relative to the parent. Figure 2D shows bifurcation angle between a parent branch and its minor daughter (angle 2) as a function of generation. It can be noticed that the angle 2 values were around 40–55 degrees for both in and out‐of‐plane bifurcation. The mean angle 2 for in‐plane bifurcation along with 95% confidence interval is 41.44 ± 1.93 degree while the mean angle 2 for out of plane bifurcation with 95% confidence interval is 41.37 ± 1.43 degree (Fig. 2).

**Figure 3:**
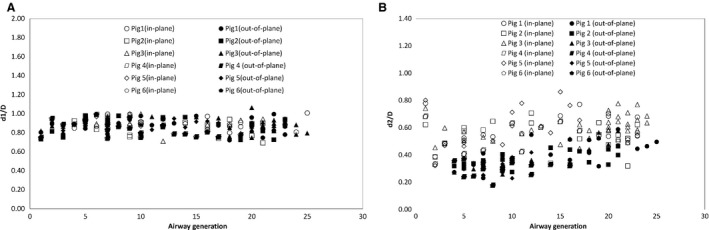
Daughter to parent diameter versus generation (A) major daughter (d1/D) ratio and (B) minor daughter ratio (d2/D).

Figure 3 shows the diameter ratio d1/D and d2/D as a function of generation. Here, D, d1 and d2 are the diameters of parent branch, major and minor daughters, respectively. The mean d1/D (with 95% confidence interval) was 0.88 ± 0.014 and 0.86 ± 0.014 for in‐plane and out‐of‐plane, respectively. The mean d2/D was, however, significantly different for in‐plane and out‐of‐plane bifurcations (*P* < 0.05, *t*‐test), especially for generations lower than 11. Here, the mean d2/D (with 95% confidence interval) was 0.57 ± 0.018 and 0.35 ± 0.022 for in‐plane and out‐of‐plane bifurcations, respectively (Fig. 3).

**Figure 4:**
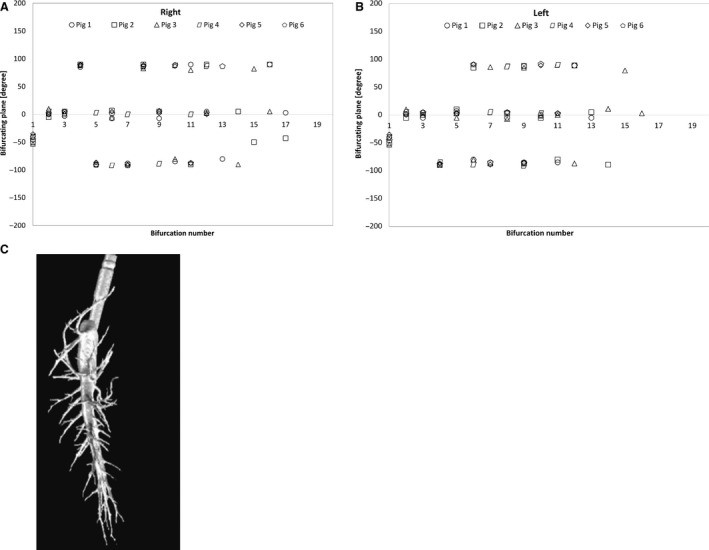
(A, B) Change in bifurcating plane of parent branch of right and left mainstem bronchi. (C) Side view of the Airway Tree.

The plane where the parent branch bifurcates into major and minor daughters can be described as the bifurcating plane. This bifurcating plane changed for in‐plane (approximately in the plane of the 2 mainstem bronchi) and out‐of‐plane bifurcation (out of that plane). Figure 4 shows the change in bifurcating plane starting from the trachea for right and left mainstem bronchi. The horizontal axis represents bifurcation number starting from the trachea, whereas the vertical axis is the angle of rotation of bifurcating plane from plane of trachea and mainstem bronchi. In this study, it is found that most of the bifurcating plane angle changed from 0, 90, and −90 degree (i.e., two consecutive out‐of‐plane in opposite directions) from the plane of the trachea and mainstem bronchi. In a few cases, only one out of plane bifurcation occurred resulting the rotation of the plane following a pattern of 0 degree then −90 degree followed by 0 degree again. Figure 4C shows that while some of the minor daughters stayed in the same plane as their parent branch plane, other minor daughters were at a plane perpendicular to the parent branch planes (Fig. 4).

**Figure 5:**
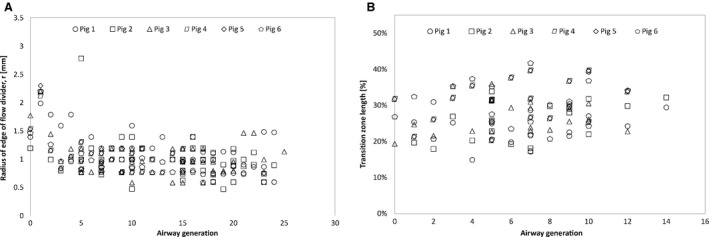
(A) Radius of edge of flow divider. (B) Percentage of transition zone at different generations.

When a parent airway branches into two daughters, the parent cross section starts as approximately circular then it transitions into an approximately elliptical cross section (Horsfield et al. 1971). This cross section varying region can be called transition zone/region. The length of the transition zone/region was approximately between 15% and 40% of the parent length for the range of generation under consideration as shown in Figure 5B.

In addition, as the two daughters originate a flow divider is formed between them. The leading edge of the flow divider is rounded with a radius, *r*, shown in Figure 5A. This radius was found to be approximately between 0.5 and 1.5 mm in this study. The two daughters may also curve before becoming straight. A radius of curvature of the daughters can be measured to describe this curving nature of airways. In this study, most of the branches had very large radius of curvature (i.e., they were mainly straight). One exception was the left mainstem bronchus which had radius of curvature of approximately 45 mm, which may be contributed to the cardiac structures (Fig. 5).

## Discussion

### Image segmentation

To extract the airway geometry, the pig lungs underwent CT scans and the resulting images were analyzed using software segmentation tools. It is to be noted that not all small airways could be found using CT imaging and automatic segmentation. While executing the volume filling algorithm, leakage from inside to outside the airways can take place and the software was stopped while executing the volume filling algorithm. Missing airways would cause errors by reducing the number of airways at each generation. This is especially true for smaller airways that are not a main focus of this article. Correction to the number of small airways can be done using methods that involve examining the terminating distal ends of airways (Tawhai et al. 2004). This was not needed in this study as it focused on the larger airways.

### Validation of segmentation

The software used for image segmentation in this study has been previously validated (Yushkevich et al. 2006). To further quantify the accuracy of segmentation, plastic tubing (with diameters 20, 12, 7, and 3 mm) were scanned with the lungs and segmented using the same procedures (automatic and manual, separately). The actual diameters of the tubing were measured using a digital caliper (0.01 mm resolution). These diameters were then compared with those provided by the segmentation software. The difference between actual and segmented measurements (both manual and automatic) was found to be less than 0.3 mm (*P* < 0.05, *t*‐test).

### Assigning generation

In order to assign a generation value to an airway, the branch diameters were sorted in descending order. The airway generation started from trachea being generation zero then the highest next diameter (e.g., right mainstem) is assigned generation 1. This procedure was followed up to generation 10 as the difference in diameter between two consecutive generations was significant (i.e. >0.3 mm). After generation 10, a different approach was taken when the difference in diameter was small (i.e. <0.3 mm). Here, instead of assigning a single diameter, a range of diameters were chosen to be a certain generation. Here, if the difference in diameter in a group of branches was 0.3 mm, they were labeled with the same generation number. In this study, similar branches across specimens were assigned the same generation. This is especially true for the vast majority of branches with d > 4 mm. This method of generation assignment helped to keep generation consistency and facilitated comparisons between specimens.

### Effect of CT resolution and diameter cut off in assigning generation

The CT used in this study has a resolution of ~0.8 mm. The resolution uncertainly associated with these measurements can then be taken as ±0.4 mm (Figliola and Beasley 2011). Note also that when creating the 3D solid model from the CT scan, the resulting surface is smooth and does not have sudden jumps of 0.8 mm, which may lead to improved surface finish and branch dimensions. It is also worth mentioning that results were not very sensitive to increasing this diameter cut off from 0.3 to 0.4 mm (e.g., the maximum number of generations will decrease by only 1–2 generations if cutoff is increased from 0.3 to 0.4 mm). Hence, a diameter cutoff of 0.3 mm was chosen.

### Pig airway morphology

The variation in branch diameter with generation (Fig. 2A) showed a similar trend to the human airways reported by Horsfield and Cumming (1968). According to Horsfield, the log mean diameter of airways dropped steeply from order 25 to order 15 (equivalent to generation 0–11) followed by a more gradual drop from order 15 to 8 (equivalent to generation 12–20). Finally, the branch diameter dropped steeply again from order 7 to 0 (equivalent to generation 21–27).

The morphology of the pig airways (Fig. 1) appeared to be significantly different from that of humans. On the other hand, several studies have shown similar morphology in pigs and other animals (Tawhai et al. 2004; Henry et al. 2014; Judge et al. 2014; Monteiro et al. 2014; Peng et al. 2015; Mansy et al. 2015a). For example, there is an early airway branching (the tracheal bronchus) that feeds the top right lung lobe that is not usually seen in humans. In addition, the two main bronchi showed a monopodial morphology, where they tended to continue in the same general direction for several generations. In this morphology, a parent branch bifurcated to a minor daughter with a relatively smaller diameter and a larger branching angle compared to the major daughter. The major daughter tended to stay in the same plane of previous major daughters. Major daughters also mostly continued in the same general direction of their parent with small deflection in direction. Many out‐of‐plane minor daughters had small diameters and large angles. The corresponding major daughters sometimes had diameters that are indistinguishable from the parent and, hence, would belong to the same generation as the parent. Daughters with same generation as parent occurred rather infrequently and were seen for generations between 5 and 25. The variation in branch diameter was small in each lung specimen for any given generation as the assigning of generations was solely based on branch diameter. Branches in a certain generation may have similar diameter with dissimilar branch length, leading to variability in branch length at each generation as seen in Figure 2B. This variation in branch length was also reported in dogs (Horsfield et al. 1982).

Moreover, the major daughter/parent diameter ratio (d1/D) for humans was reported to be 0.89 (Horsfield and Cumming 1968), which is comparable with pig airways value of 0.87 found in this study. The minor daughter to parent diameter ratio (d2/D) showed bimodal character at least up to generation 10. This phenomenon is not evident in human bronchial tree which is bipodial (Kabilan et al. 2007) or dichotomous (Horsfield and Cumming 1968) in nature and has a d2/D value around 0.66 (Farag 1998). In this study, d1/D for in‐plane and out‐of‐plane case found to be significantly different than d2/D (*P* < 0.05, *t*‐test). Overall, the vast majority of bifurcations observed in this study were asymmetric. Similar trends were seen in the dog (Horsfield et al. 1982) and pig (Maina and Gils 2001). In addition, bifurcation angles for minor and major daughters were significantly different (*P* < 0.05, *t*‐test) for both in‐plane and out‐of‐plane branches.

### Effect of breathing phase on the measured dimensions

The airway dimensions present in this article relied on CT scans that were acquired close to full inspiration. Hence, the extracted geometry would correspond to the largest airway dimensions during the breathing cycle. The airway dimensions are expected to change during breathing. The lung volume was reported to vary by approximately 26% during breathing in humans (Horsfield et al. 1971). To explore changes in the airway dimensions during breathing, a CT scan of one lung was performed at full expiration and the diameter of airways as small as 3 mm was measured. Measurements showed that the changes in the airway diameter were undetectable (i.e., less than the image resolution of 0.8 mm. The volume of the parenchyma showed significant changes as expected. The small changes in airway diameters may be due to the relatively high cartilage content of large airways that increased their stiffness. Smaller airways have less stiff walls but have small diameters and possibly small diameter changes, which might have made these changes undetectable. For example, a diameter change of 0.8 mm for a 3 mm airway would constitute >25% diameter change. It can then be concluded that diameter changes >25% during the breathing cycle were not seen in this study.

### Effect of airway morphology in airflow inside airways

The monopodial morphology of the bronchial tree has a great significance on airflow. It is to be noted that for the first 10 generations, the major daughter direction was close to the parent direction and the minor daughters tended to grow laterally, which lead to the current monopodial nature. This airway morphology is associated with smaller airflow deflection for major daughters which would tend to enhance flow rates in the major daughters compared to the minor daughters. This would also tend to inhibit the formation of Dean's flow in the major daughters (Gamage PT: Private communication). In this study, most minor daughters grew laterally from the left and right mainstem bronchi and there was a small number of minor daughters that grew medially as can be seen in Figure 1.

### Rotation of bifurcating plane

In addition, major daughters (up to at least the 10th generation) tended to stay in the same approximate plane of the mainstem bronchi. While some minor daughters stayed in that same plane, other daughters tended to bifurcate into the perpendicular plane. The in‐plane and out‐of‐plane bifurcations occurred in an alternating manner where for each in‐plane bifurcations, the next two bifurcations were often out‐of‐plane in opposing directions. For example, minor daughters in the mainstem bronchi (both left and right) were in the same plane for the 1st, 4th and 7th bifurcations, and in the perpendicular plane for the 2nd, 5th, and 8th bifurcations. The 3rd, 6^th^, and 9th were also in the perpendicular plane but growing in the opposite directions.

### Radius at the flow divider and transition zone

In this study, the radius of flow divider was 0.5–1.5 mm, which was comparable to previous studies (Horsfield et al. 1971). Transition zone as a percentage of branch length was slightly longer than values reported for human airways (Farag 1998). The current measurements showed that the airway cross sections outside the transition zone were almost cylindrical as the aspect ratio (D_min_/D_max_) ranged from 0.9 to 0.98 for the airways considered in the study. On the other hand, the cross section was noticeably noncircular in the transition zone as expected.

### Effects of different techniques in creating physical model of airway tree

This study used computed tomographic imaging along with segmentation software tools to extract information on the dimensions and angles of the pig airways. This technique has advantages over the traditional preparation of lung casts, where, for example, elastomers are injected through the trachea and airways as the latter method cannot be done in live species. This resulting cast will also likely depend on the properties of the materials used, for example: the amount of shrinkage after curing, the time it takes to settle, the viscosity of the material, etc. Furthermore, the bronchial tree is recovered by dissolving cast into an acid or base solution which eventually destroys the lung by dissolving all tissue in the solution. The casting process also takes considerable amount of time compared to extracting the geometry from the CT scan of the organ, which can be reproduced within short time and provides a geometry that can be directly used in computer simulations of, for example, flow in the airways.

### Study limitations

To document airway morphology, this study used preserved lungs (LS03765, BioQuest, NASCO, Fort Atkinson, WI) with elastic properties that may vary from lungs in living animals. Although there are no available direct measurements of lung morphology preservation of these lungs, previous studies that used this type of lungs suggested proper preservation levels (Graf et al. 2009; Ihra et al. 2010). To further check for lung and airway elasticity, air was injected in the trachea and air pressure and volume were recorded. Data showed that a pressure of 10 cm of water was sufficient to fully inflate the lungs (with 2‐L tidal volume), which suggests proper lung elasticity. Note also that one of the preserved lungs was later dissected and no evidence of airway disintegration or stiffening was found. In addition, this study has focused on larger airways that tend to maintain their dimensions due to their relatively high cartilage content.

## Conclusions

Geometric information, such as dimensions and angles, of the pig airways is needed when constructing physical models of the pig airway tree or when performing computer simulations of flow or sound transmission in the pig airways. There is little information on the pig airway geometry in the literature. This study extracted information on the length, diameter and bifurcation angles of the airways using computed tomographic imaging along with segmentation software tools. Results showed that the geometric features of the pig airways can be significantly different from those of humans. This suggests that when constructing realistic computer models of the pig chest, the human geometry may not provide a good approximation. The use of actual pig airway geometry (instead of the human airway approximation) for physical models or in numerical simulation of airflow and sound transmission in the pig airways would result in constructing more realistic physical and computational models and possibly more accurate measured and simulation results.

## Conflict of Interest

No conflicts of interest, financial or otherwise, are declared by the author(s).
